# Supplementary material for: Splice-Junction-Based Mapping of Alternative Isoforms in the Human Proteome
Source: Cell Rep. Author manuscript; Available in PMC 2020 Jan 15. (PMC6961840; doi:10.1016/j.celrep.2019.11.026)
Supplement: 2 [file NIHMS1546469-supplement-2.zip › DF1/PXD010154/Liver/Liver_10_HP_TEGDDDGCPKPPEIAHGYVEHSVR.pdf]

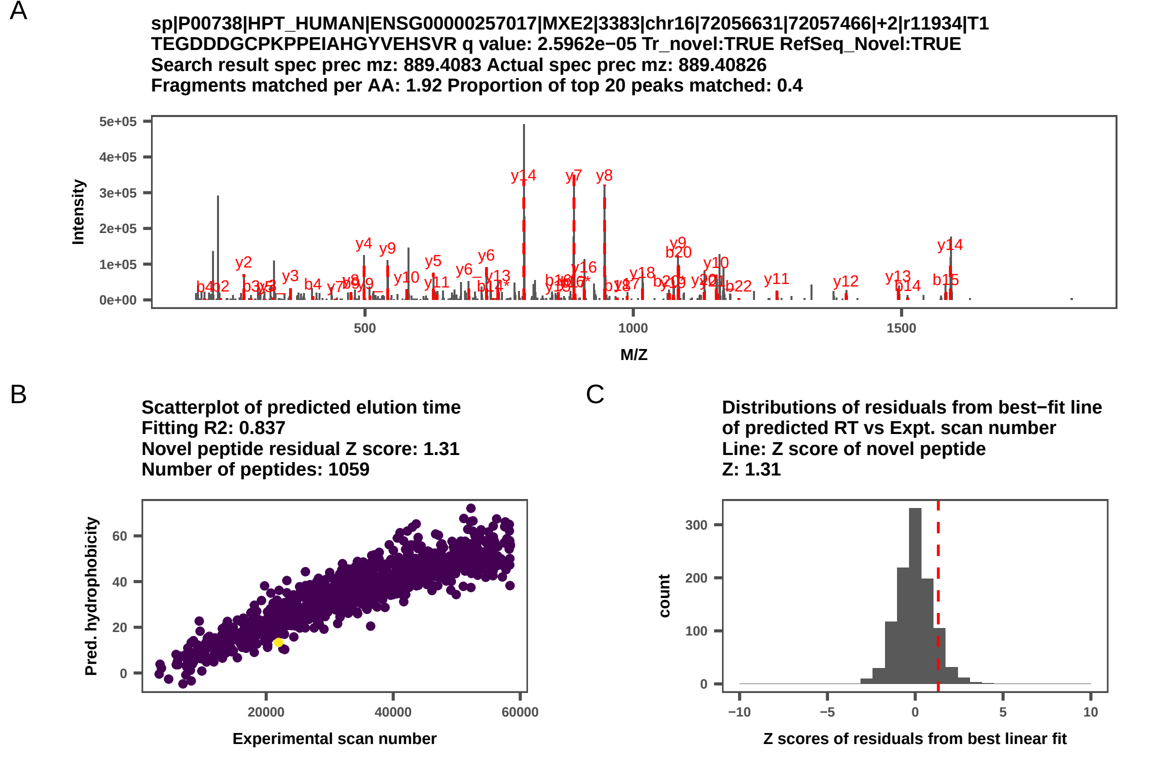
A
sp|P00738|HPT_HUMAN|ENSG00000257017|MXE2|3383|chr16|72056631|72057466|+2|r11934|T1
TEGDDDGCPKPPEIAHGYVEHSVR q value: 2.5962e−05 Tr_novel:TRUE RefSeq_Novel:TRUE
Search result spec prec mz: 889.4083 Actual spec prec mz: 889.40826
Fragments matched per AA: 1.92 Proportion of top 20 peaks matched: 0.4
Intensity
5e+05
4e+05
3e+05
2e+05
1e+05
0e+00
500
1000
1500
M/Z
y14
y7
y8
y4
y9
y2
y3
y5
y6
y10
y11
y13
y16
y18
y9
b20
y10
y11
y12
y13
b14
y14
b15
b22
b4
b2
b4
B
Scatterplot of predicted elution time
Fitting R2: 0.837
Novel peptide residual Z score: 1.31
Number of peptides: 1059
Pred. hydrophobicity
60
40
20
0
20000
40000
60000
Experimental scan number
C
Distributions of residuals from best−fit line
of predicted RT vs Expt. scan number
Line: Z score of novel peptide
Z: 1.31
count
300
200
100
0
−10
−5
0
5
10
Z scores of residuals from best linear fit
